# Possible Use in Soil Bioremediation of the Bacterial Strain Bacillus Sphaericus NM-1 Capable of Simultaneously Degrading Promethrin and Acetochlor

**DOI:** 10.3390/microorganisms13071698

**Published:** 2025-07-19

**Authors:** Yue Cheng, Qian Fu, Junjia Xu, Xinhua Niu, Lin Liu, Jiaqi Wang, Jingwen Quan, Qingyue Yu, Baoyan Chi, Haitao Li, Rongmei Liu

**Affiliations:** 1 Heilongjiang Green Food Science Research Institute, Harbin 150023, China; chengyue1523@163.com; 2 College of Life Sciences, Northeast Agricultural University, Harbin 150030, China; fuqian9612@163.com (Q.F.); 13922731677@163.com (J.X.); niuxinhua0519@163.com (X.N.); 15164687829@163.com (L.L.); 19845921897@163.com (J.W.); jeongmoon0000@163.com (J.Q.); yuqingyue1991@neau.edu.cn (Q.Y.); chibaoyan@neau.edu.cn (B.C.)

**Keywords:** prometryn, acetochlor, microbial bioremediation, soil enzyme activity, soil microbial community, soybean seedlings

## Abstract

Prometryn and acetochlor are herbicides used to control weeds in farmlands and other areas. They enter the soil through direct application, residual accumulation in crops, and atmospheric deposition. The pollution of their residues in the environment has attracted people’s attention. Bioremediation is one of the main methods to solve such problems. In this study, the effects of prometryn and acetochlor-degrading strain NM-1 on soil enzymes, soil microbial communities, and physiological indexes of soybean seedlings during soil remediation were studied, and the relationship between them was discussed. The results showed that 81.54% of prometryn (50 mg·L^−1^) and 89.47% of acetochlor (50 mg·L^−1^) were degraded within 15 days after NM-1 inoculation in soil. NM-1 positively affected soil enzyme activities and soil microbial communities, and the abundance of beneficial bacteria in soil increased. More importantly, the inoculation of strain NM-1 under prometryn and acetochlor stress significantly increased plant height, root length, root volume, water content, chlorophyll concentration, and root activity of soybean. The results of these studies showed that the NM-1 strain showed significant potential in bioremediation in order to provide technical support for solving the problem of prometryn and acetochlor pollution.

## 1. Introduction

Herbicides work to control weeds and are usually used in agricultural crops, which increases the grain production tremendously in the world [1]. Due to the limited effectiveness of single herbicides, many commodities are now mixtures, which have better weed control and are applied over a wide range [2]. However, the extensive use of herbicides has a negative impact on the ecosystem. As of 2021, the global use of triazine herbicides is about 9021 tons [3]. Among them, prometryn, one of the triazine herbicides, is widely used and highly stable, which makes prometryn accumulate in crops and soil, and even contaminate rivers and aquatic organisms [4]. Studies have shown that the growth of seedlings of *Phaseolus vulgaris* L. was inhibited and the accumulation of photosynthetic products was reduced when the soil was treated with high concentrations of prometryn [5]. Some scholars believe that prometryn has a certain negative impact on the body length, heart, and color perception of zebrafish, and can even induce DNA damage in human bronchial epithelial cells [3,6,7]. Acetochlor is a chloroacetanilide herbicide that is used in more than 10,000 tons in China [8]. Some studies have shown that acetochlor can cause damage to photosynthetic organs and a reduction in photosynthetic rate in grape leaves [9]. Zhang and Wang et al. reported that high doses of acetochlor disrupted the development of zebrafish ovaries and induced developmental defects in zebrafish [10,11]. More importantly, acetochlor can increase the risk of cancer and is hereditary [12]. Prometryn and acetochlor are extremely harmful, so it is necessary to remove residues in the environment, and microbial degradation is the most economical and environmentally friendly method [13]. In our laboratory, a strain was screened in polluted soil for degradation of prometryn and acetochlor, which was able to degrade more than 60% of prometryn and more than 80% of acetochlor within 7 d at a concentration of 100 mg·L^−1^, and was identified as *Bacillus sphaericus* named NM-1 by 16SrRNA sequencing and physiological indexes. The study showed that *Bacillus* has an important role in agroecosystems, not only improving polluted soil but also positively affecting plant growth [14,15]. However, studies on prometryn- and acetochlor-degrading bacteria have mainly focused on their degradation pathways [16,17,18]. Therefore, in this study, we will proximally assess the role of this strain in the bioremediation of prometryn- and acetochlor-contaminated soils through soil enzymes, soil microbial communities, and effects on soybean seedlings.

Soil enzymes and microbial communities are important indicators of soil conditions; they are involved in various biochemical processes in the soil and play an important role in soil metabolism, affecting the process of material exchange and transformation and nutrient decomposition and release within the soil [19,20]. The change in soil enzyme activity can sensitively reflect the change in soil quality, which is a biological index to measure soil quality and soil biological activity [21,22]. It has been shown that Butisanstar and Clopyralid inhibit dehydrogenase and catalase activities in soil even at the lowest dose [23]. Zhang et al. also indicated that atrazine affects the activities of catalase, cellulase, urease, and sucrase in the soil, and strain ZF-1 could mitigate the effect of atrazine contamination on the activities of these four enzymes [24].

Soil microorganisms play an important role in carbon cycling as well as the bioremediation of soil pollutants and biocontrol of soil–plant pathogens [25,26]. Some studies have shown that the abundance and number of soil microbial communities changed positively after inoculation of the *Chenggangzhangella methanolivorans* CHL1 strain in metsulfuron-methyl- or tribenuron-methyl-contaminated soils, which is more favorable to the growth of plants in the soil [27]. In this study, soil enzymes were used as indicators to study the effects of the introduction of exogenous degrading bacteria on crops, soil enzymes, and soil microorganisms by observing the changes in soil microbial communities and soybean physiological indicators, and the bioremediation effect of the introduction of exogenous degrading bacteria on contaminated soil was further analyzed.

Therefore, we measured the changes in soybean seedlings, soil microbial communities, and enzyme activities (sucrase, urease, cellulase, and catalase) by soybean physiological indexes detection, soil enzyme activity determination, and observation of soil microbial community structure by high-throughput sequencing, aiming to study the potential effects of *Bacillus sphaericus* on soil ecology in the process of soil remediation. The results of this study can enrich the resources of bacterial strains, fill the gaps of the effects of exogenous prometryn- and acetochlor-degrading strains on soil enzyme activities, expand the understanding of the effects of degrading strains on the structure of bacterial communities, broaden the understanding of the changes in exogenous degrading strains on the soil ecological environment, and provide a guide for the in situ remediation of prometryn- and acetochlor-contaminated soils.

**Figure 1:**
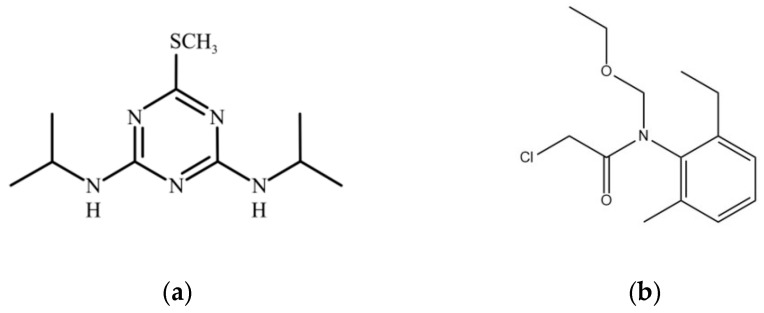
(**a**) Chemical structure of prometryn; (**b**) chemical structure of acetochlor.

To comprehensively understand the behavior of prometryn and acetochlor in soil and various environmental compartments, presenting their key chemical and physical properties is crucial. The following table details these properties, including the chemical structures, along with relevant references for each value, as shown in Figure 1a,b and Table 1.

## 2. Materials and Methods

### 2.1. Experimental Material

#### 2.1.1. Main Chemical Reagents and Culture Media

Prometryn (>95%) and acetochlor (>95%) were purchased from Beijing Qincheng Yixin Technology Development Co., Ltd. (Beijing, China).

LB medium.

Trypton: OXOID (Thermo Fisher Scientific), Leicestershire, UK.

Yeast extract: OXOID (Thermo Fisher Scientific), Leicestershire, UK.

NaCl: Tianjin Damao Chemical Reagent Factory, Tianjin, China.

Agar: Shanghai Aladdin Biochemical Technology Co., Shanghai, China.

Inorganic salt medium.

Distilled water.

K_2_HPO_4_: Tianjin Benchmark Chemical Reagents Co., Tianjin, China.

KH_2_PO_4_: Tianjin Benchmark Chemical Reagents Co., Tianjin, China.

MgSO_4_·H_2_O: Tianli Chemical Reagent Co., Shanghai, China.

CaCl_2_·H_2_O: Tianjin Benchmark Chemical Reagents Co., Tianjin, China.

FeSO_4_: Tianjin Zhiyuan Chemical Reagent Co., Tianjin, China.

Na_2_MoO_4_: Tianjin Chemical Reagents No.4 Factory, Tianjin, China.

#### 2.1.2. Experimental Soil Samples

Screened soil sample: The soil sample was collected from a farmland (43°22′00.00″ N, 121°42′00.00″ E to 43°58′00.00″ N, 123°02′00.00″ E) in Horqin District, Tongliao City, Inner Mongolia Autonomous Region, which was contaminated by multiple compound pesticide agents. After sampling, the sample was subjected to a 30-mesh sieving treatment, and then stored in plastic bags and kept at 4 °C.

Soil sample for bioremediation: The soil from the experimental field of Northeast Agricultural University without pesticide contamination was air-dried and ground, and then passed through a 30-mesh sieve and stored at 4 °C.

#### 2.1.3. Biochemical Reagent

The biochemical reagents used in this study, including those for molecular cloning, enzyme digestion, and physiological indicator detection, were sourced from various manufacturers. Detailed information on these reagents, such as the product names, suppliers, and their respective countries, is provided in Table 2.

#### 2.1.4. Experimental Instruments and Equipment

A variety of instruments were employed to carry out the experiments, spanning from sample processing and cultivation to detection and analysis. The specific models, manufacturers, and sources of these instruments are summarized in Table 3.

### 2.2. Soil Properties

The test soil (pesticide-free) used in the pot experiment was collected from the experimental field of Northeast Agricultural University (Harbin, China). Prior to the experiment, basic physicochemical properties of the soil were analyzed using standardized methods:

pH: Measured potentiometrically in a 1:2.5 (*w*/*v*) soil-water suspension using a pH meter (Model PHS-3C, Shanghai INESA Scientific Instrument Co., Ltd., Shanghai, China), with a value of 6.5 ± 0.3.

Texture: Determined by the pipette method, consisting of 18% clay (<2 μm), 35% silt (2–50 μm), and 47% sand (50–2000 μm), classified as sandy loam.

Organic Matter (OM%): Quantified via the Walkley–Black oxidation method, with a content of 2.1 ± 0.2% (dry weight basis).

Carbonates: Analyzed by acid neutralization (using 0.1 M HCl) and expressed as CaCO_3_ equivalent, with a low content of 0.2 ± 0.05%.

Cation Exchange Capacity (CEC): Measured by the ammonium acetate method (pH 7.0), yielding a value of 12.3 ± 0.8 cmol(+)/kg.

Water Holding Capacity (WHC): Determined by saturating soil samples and allowing drainage to field capacity, with a maximum WHC of 28 ± 1.5% (mass water content).

These properties are relevant to interpreting the degradation efficacy of prometryn and acetochlor:

The sandy loam texture (moderate clay and sand content) and moderate OM% likely balanced pollutant adsorption (reducing bioavailability) and microbial activity (supporting strain NM-1 growth).

The neutral pH (6.5) and low carbonate content minimized pH-induced changes in compound speciation, ensuring consistent bioavailability.

The CEC and WHC values indicate the soil’s capacity to retain moisture and nutrients, which may have supported stable microbial degradation over the experimental period.

By including these details, we provide a clear context for how soil characteristics could have conditioned the observed results, enhancing the interpretability and reproducibility of our findings.

### 2.3. Preparation of Soil Samples and Bacterial Suspensions

The soil used in the experiment was taken from the experimental field of Northeast Agricultural University to ensure that it was not polluted by prometryn and acetochlor. The soil was air-dried, ground, passed through a 2 mm sieve, and divided into two parts. The first part was the fresh soil without sterilization. Prometryn and acetochlor (prometryn and acetochlor dissolved in methanol) were artificially added and sprayed evenly to make the concentration of prometryn and acetochlor in the soil 100 mg·kg^−1^, respectively. Then the soil mixture was dried in a fume hood until the methanol was completely volatilized. In the second part of the experiment, the soil was sterilized (121 °C, 50 min), and the treatment of the first part was repeated.

The strain NM-1 was enriched and cultured in MSM medium containing prometryn and acetochlor. The cells were grown to OD600 = 1.0, collected, and washed three times in sterile water. The enriched culture was resuspended in sterile water to obtain a cell density of about 1 × 10^8^ CFU·mL^−1^.

### 2.4. Experimental Design and Treatment

The strain NM-1 was inoculated into LB medium (containing tryptone, yeast extract, sodium chloride, distilled water, and 100 mg·L^−1^ of prometryn and acetochlor) for enrichment culture. The bacteria were collected when the cells grew to OD600 = 1.0, washed 3–5 times with sterile water, and resuspended with sterile water to a bacterial concentration of about 1 × 10^8^ CFU·mL^−1^. The experimental treatments were as follows: (A) sterilized soil (200 g) + sterile water (10 mL); (B) sterilized soil (200 g) + NM-1 bacterial solution (10 mL, 1 × 10^8^ CFU·mL^−1^); (C) fresh soil (200 g) + sterile water (10 mL); (D) fresh soil (200 g) + NM-1 bacterial solution (10 mL, 1 × 10^8^ CFU·mL^−1^), and each treatment group was repeated three times. The experiment was conducted at room temperature with daily water spraying to ensure that the humidity of the soil was around 70%, and samples were taken every 3 days until the 15th d of treatment, with 10 g of samples taken each time to detect the residual amount of prometryn and acetochlor in the soil, which was used to test the remediation effect of strain NM-1 on prometryn and acetochlor soil.

### 2.5. Extraction and Detection of Prometryn and Acetochlor

The extraction of prometryn and acetochlor in soil: 10 g of the soil sample to be tested was weighed, to which 20 mL of dichloromethane was added, and the sample was shaken vigorously in a shaker for 1 h, then left to stand for 1 h, and centrifuged at 12,000 r·min^−1^ for 15 min. The 10 mL supernatant was concentrated and evaporated using a rotary evaporator. The remaining prometryn and acetochlor were dissolved in 2 mL chromatographic grade methanol and filtered with a 0.22 μm organic phase filter membrane. The filtrate (Jiangsu Huida Medical Instruments Co., Ltd., Yancheng, China) was stored in a liquid phase bottle. Finally, the content of prometryn and acetochlor was determined by HPLC [28].

A high-performance liquid chromatography (Agilent 1260 Infinity II, Santa Clara, CA, USA) equipped with a reversed C18 column (4.6 × 100 mm, 5 μm) was used to detect the residues of prometryn and acetochlor. The HPLC detection conditions of prometryn and acetochlor were as follows: the injection volume was 10 μL, the flow rate was 1.0 mL·min^−1^, the column temperature was 35 °C, the detection wavelength was 215 nm, and the mobile phase was acetonitrile–water = 70:30 (*v*:*v*).

According to the residues of prometryn and acetochlor, the degradation rate of prometryn and acetochlor was calculated. The calculation formula of the degradation rate was as follows:

$$X=\frac{\mathrm{CCK}-\mathrm{CX}}{\mathrm{CCK}}\times 100\%$$

In the formula, X is the degradation rate of prometryn and acetochlor; CX is the final concentration of prometryn and acetochlor; CCK is the initial concentration of prometryn and acetochlor [29].

### 2.6. Pot Experiment Setup and Treatment

The pot experiment was conducted using clean soil (pesticide-free) from the experimental field of Northeast Agricultural University as the substrate. The soil was air-dried, ground, and sieved through a 30-mesh sieve to remove debris. Soybean seedlings with uniform and healthy growth were transplanted into potting buckets (5 cm in diameter), with each bucket containing 0.5 kg of sifted dry soil.

Irrigation was performed regularly to maintain consistent soil moisture across all the treatments, monitored using a soil moisture meter. Deionized water was used for irrigation to avoid introducing external contaminants. Seeds of the test plant were purchased from Heilongjiang Academy of Agricultural Sciences, Harbin, China, with a germination rate ≥ 95%.

The experiment was carried out in an artificial climate chamber (Yiheng Scientific Instruments Co., Ltd., Shanghai, China) under controlled environmental conditions: photoperiod of 16 h light/8 h dark, light intensity of 300 μmol·m^−2^·s^−1^, temperature maintained at 25 ± 1 °C during the day and 18 ± 1 °C at night, and relative humidity of 70 ± 5%.

Three treatments were set up in this experiment:

(1) Control group (CK): Soil in the potting buckets was not inoculated with microorganisms and not sprayed with pesticides.
(2) Treatment group 1 (PA): Mother liquor of prometryn and acetochlor was sprayed evenly into the potting buckets until the final concentration of both prometryn and acetochlor in the soil reached 50 mg·kg^−1^.
(3) Treatment group 2 (NMJ): Potting buckets were evenly sprayed with prometryn and acetochlor mother liquor to a final concentration of 50 mg·kg^−1^ for each pesticide, followed by the addition of a bacterial suspension of strain NM-1 (concentration: 1 × 10^8^ CFU·mL^−1^), which was mixed thoroughly.

Each treatment had 30 replicates, and the experimental setup followed the guidelines outlined in “Standardized Methods for Soil Bioremediation Pot Experiments” (GB/T 36197-2018 [30]) to ensure reproducibility and comparability of the results.

Soil samples were collected at 7 d, 14 d, 21 d, and 28 d after application using a random multi-point sampling method. The collected soil samples were divided into two parts: one for analyzing soil enzyme activity, and the other for assessing changes in microbial community structure (the sample names were abbreviated as CK, PA7, NMJ7, PA14, NMJ14, PA21, NMJ21, PA28, and NMJ28 for microbial community analysis).

Additionally, the root length, plant height, water content, root volume, chlorophyll content, carotene content, and root activity of soybean seedlings were measured on the 7th, 14th, and 21st days after application [31].

### 2.7. Detection of Physiological Indexes of Soybean Seedlings

Soybean root activity was detected using the TTC method, and chlorophyll was extracted from seedlings using the ethanol method [32].

### 2.8. Determination of Soil Enzyme Activities

The activities of soil sucrase, cellulase, urease, and catalase were determined by 3,5-dinitrosalicylic acid colorimetry, anthrone colorimetry, phenol sodium–sodium hypochlorite colorimetry, and the ultraviolet absorption method, respectively [33,34,35].

### 2.9. Soil DNA Extraction and High-Throughput Sequencing

DNA was extracted from soil samples, and the extracted DNA was analyzed qualitatively and quantitatively to detect the quality of DNA. The V3-V4 variable region of soil microbial genome 16S rDNA was amplified using primers 347F (5′-CCTACGGRRBGCASCAGKVRVGAAT-3′) and 802R(5′-GGACTACNVGGGGTWTC TAAT CC-3′) [24]. The reaction system consisted of 25 μL 2× Rapid Taq Master Mix, 2 μL Primers-f, 2 μL Primers-r, 100 ng Genomic DNA, and ddH2O, which made up to 50 μL. Amplification was performed for 32 cycles after pre-denaturation at 94 °C for 2 min. Cycling conditions were set to denaturation at 94 °C for 15 s, annealing at 59 °C for 30 s, and extension at 72 °C for 90 s. Nucleic acid gel verification was run further and the AxyPrep DNA Gel Extraction Kit (Axygen Scientific, Inc., Union City, CA, USA) was used to purify the product, and then the purity of the purified product was analyzed and quantified. The raw image data files acquired through Paired-End sequencing (utilizing the Illumina MiSeq™/HiSeq™ sequencing platforms, San Diego, CA, USA) were analyzed via Base Calling to convert them into raw sequences. After obtaining these, the primer junction sequences were first removed. Subsequently, the pairs of reads were merged into a single sequence based on the overlap relationships among PE reads. According to the sequences of the barcode tags, sample differentiation and identification were then conducted, leading to the processing of the sample data. Finally, quality control measures were implemented to ensure the validity of the data for each sample. Through the clustering operation of the Usearch software11.0 (Herzliya, Israel), the sequences were divided into many groups according to their similarity, and one group was an OTU. According to different similarity levels, all sequences can be divided into OTUs, and the biological information statistical analysis of OTUs at a 97% similarity level can be carried out to obtain the representative sequences of OTUs, and the species classification of each group can be obtained according to the sequence composition of OTUs.

### 2.10. Statistical Analysis

QIIME2 2023.9.2 (Zurich, Switzerland) and R software4.3.1 (Auckland, New Zealand) were used to analyze the species composition of the soil samples, and Beta diversity analysis was used to compare the differences in the species community structure and composition of the soil samples in different treatment groups; redundancy analyses were performed using CANOCO 5.0 (Beijing, China) to assess the relationship between environmental factors and dominant species of bacterial communities, and double-matrix correlation analyses were performed to explore the relationships among the dominant species, soil enzymes, and bacterial strains of the soil.

One-way analysis of variance (ANOVA) was used to analyze significant differences between treatment samples, with *p* < 0.05 indicating statistically significant differences, and GraphPad Prism 8.0.2 (GraphPad Software, San Diego, CA, USA) was used for statistical calculations and plotting.

## 3. Results and Discussion

### 3.1. Bioremediation of Prometryn and Acetochlor Contaminated Soil by Strain NM-1

**Figure 2:**
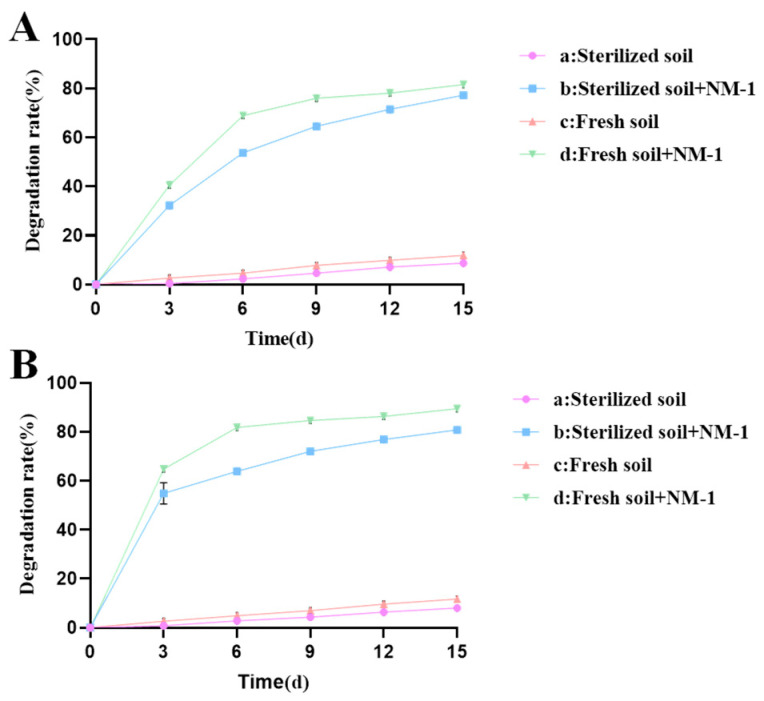
Residue of prometryn (**A**) and acetochlor (**B**) in soil.

Strain NM-1 showed good degradation in 1/2 LB medium containing prometryn and acetochlor (100 mg·L^−1^). The strain degraded about 64% of prometryn and 84% of acetochlor within 7 d. A number of strains capable of degrading prometryn and acetochlor have been reported, including strains capable of degrading prometryn, such as P-1, P-2, and JW-1, as well as strains capable of degrading acetochlor, such as *Bacillus* sp. ACD-9, *Sphingomonas chloroacetimidivorans*, *Rhodococcus* sp. T3-1, and *Delftia* sp. T3-6 [17,36,37,38], but these strains were not shown to be capable of degrading both prometryn and acetochlor. In this study, we investigated the remediation effect of strain NM-1 on prometryn- and acetochlor-contaminated soil, the ability of this strain to degrade prometryn and acetochlor in soil. The residue dynamics analyses of prometryn and acetochlor in four soil samples (a, b, c, and d) are shown in Figure 2A and B, respectively.

The results showed that after the addition of strain NM-1, the degradation rate of prometryn (50 mg·kg^−1^) in treatment groups b and d reached 71.46% and 78.06%, respectively, and the degradation rate of acetochlor reached 76.91% and 86.25%, respectively, within 12 days. In treatment groups a and c, the degradation rates of prometryn (50 mg·kg^−1^) were only 7.12% and 9.85%, and that of acetochlor were only 4.32% and 9.65% at 12 days, respectively. Therefore, strain NM-1 significantly improved the degradation efficiency of prometryn and acetochlor in soil. Zheng and Liang et al. also pointed out that the inoculation of strain NMA-1 could accelerate the degradation of butachlor in soil, and the inoculation of strain *Pseudomonas* sp. DY-1 could also accelerate the degradation of prometryn in soil [16,39], which indicated that the inoculation of degrading bacteria had a certain remediation effect on polluted soil. Treatment groups b and d were compared with each other; the degradation efficiencies of prometryn and acetochlor were higher in treatment group d than in treatment group b. Treatment groups a and c were compared with each other; the degradation efficiencies of prometryn and acetochlor were higher in treatment group c than in treatment group a, which may be due to the fact that some microorganisms in the soil promoted the degradation of prometryn and acetochlor [39,40]. In addition, the degradation rate of prometryn and acetochlor in treatment group a reached 8.68% and 8.03% at 15 d, probably due to hydrolysis or oxidation [41]. In this study, strain NM-1 showed good degradation of prometryn and acetochlor in soil. Therefore, strain NM-1 is a good candidate for the remediation of prometryn- and acetochlor-contaminated soil.

### 3.2. Effect of Strain NM-1 on Soil Enzyme Activity

**Figure 3:**
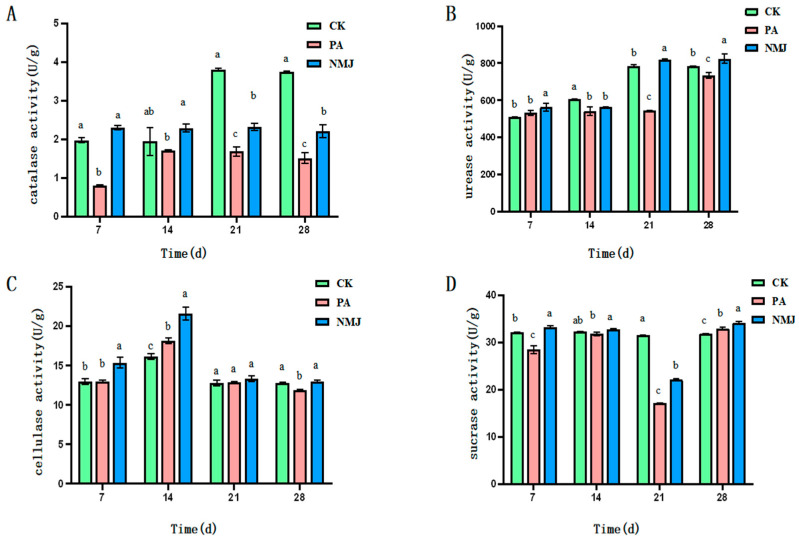
Effect of strain NM-1 on the activity of catalase (**A**), urease (**B**), cellulase (**C**), and sucrase (**D**) in soil treated with prometryn and acetochlor. (*p* < 0.05). The error bars represent the standard deviation (SD). Lowercase letters (a, b, c) above the bars indicate significant differences between treatment groups at the same time point, as determined by one-way analysis of variance (ANOVA) followed by post-hoc tests. Values with different lowercase letters are significantly different (*p* < 0.05), while those with the same lowercase letter are not significantly different.

As shown in Figure 3A, catalase activity was inhibited by prometryn and acetochlor. The application of strain NM-1 increased soil catalase activity and reduced the inhibition of soil catalase activity by prometryn and acetochlor. At the 7th and 14th d, the inhibition of soil catalase activity by prometryn and acetochlor was alleviated by strain NM-1, and the catalase activity of the NMJ group was higher than that of the PA and CK groups, with 2.30 U·g^−1^ and 2.29 U·g^−1^, respectively. Over time, the enzyme activities of the PA and NMJ treatment groups declined in comparison with that of the CK group, but the enzyme activity of the NMJ group was always higher than that of the PA group, indicating that strain NM-1 promoted soil catalase activity. It has been shown that acetochlor and atrazine inhibited catalase at a higher rate, and the enzyme activity could not be restored to the level without herbicide application after 28 d. This conclusion is consistent with the results of this study [42].

As shown in Figure 3B, prometryn and acetochlor were able to inhibit soil urease activity. Strain NM-1 increased the enzyme activity in the soil contaminated with prometryn and acetochlor. As shown in Figure 3, the urease activity was significantly reduced in the PA group compared with CK at 14 d with the addition of prometryn and acetochlor. At d 21, the enzyme activity of the NMJ group was higher than that of the PA group, with an enzyme activity of 817.778 U·g^−1^. With the continuous degradation of prometryn and acetochlor in the soil, the urease activity of the NMJ-treated group was continuously decreasing, and it recovered at 21 d. The urease activity of the NMJ group was higher than that of the PA group throughout the whole incubation process, which indicated that strain NM-1 was able to alleviate the inhibition of soil urease by prometryn and acetochlor to a certain extent. Some studies showed that acetochlor and atrazine inhibited urease at a high rate, and the enzyme activity could not be restored to the level without herbicides after 28 d. This conclusion was consistent with the results of the present study [42]. Meanwhile, the enzyme activity of the NMJ group was higher than that of the CK group at 21 d, which indicated that NM-1 could promote the urease activity.

As shown in Figure 3C, prometryn and acetochlor promoted cellulase activity in the soil, while inoculation of strain NM-1 further increased soil cellulase activity. The value of cellulase activity in the CK group was 16.10 U·g^−1^. On the 14th day of the prometryn and acetochlor application, the cellulase activity in the PA group and the NMJ group was 18.13 U·g^−1^ and 21.57 U·g^−1^, respectively. After a period of time, the cellulase activity of the PA and NMJ treatment groups gradually decreased with the decrease in the concentration of prometryn and acetochlor in the soil. At 28 d of incubation, the activity value of the PA group was lower than that of the CK group, while the NMJ group was still higher than that of the CK group, indicating that strain NM-1 could increase the activity of soil cellulase. It has been shown that cellulase improves soil fertility, and its role is thought to be mainly acting as a biocontrol agent that mediates inhibition [43]. Prometryn and acetochlor were observed to increase cellulase activity in soil, which may reflect a stress-induced response of soil microorganisms to pesticide exposure rather than acting as biocontrol agents. This elevation in cellulase activity could be a result of microbial adaptation to the presence of these herbicides, potentially aiding in the decomposition of organic matter under pollutant stress, but it does not mitigate the overall inhibitory effects of prometryn and acetochlor on organisms.

As shown in Figure 3D, prometryn and acetochlor were able to inhibit sucrase activity in soil. The inhibitory effect on soil sucrase was most obvious 21 d after the addition of prometryn and acetochlor, at which time the activity of soil sucrase in the PA group was 17.11 U·g^−1^, which was reduced by 45.7% compared with that of CK, and with the degradation of prometryn and acetochlor, the activity of soil sucrase gradually recovered. Strain NM-1 alleviated the inhibitory effect of prometryn and acetochlor on soil sucrase, and the enzyme activity of NMJ in the treatment group was higher than that of the CK group at 7 d, 21 d, and 28 d of inoculation with the strain. The study showed that acetochlor and atrazine inhibited sucrase at a higher rate, a finding consistent with the results of the present study [42]. Liu et al. showed that sucrase activity was significantly reduced in soils where atrazine was applied for a long period of time [44]. Prometryn and acetochlor showed inhibitory effects on the enzyme activity in soil, but strain NM-1 was able to restore sucrase activity in soils contaminated with prometryn and acetochlor, and soil sucrase activity continued to be restored over time and was even higher than that of the CK group.

Soil enzymes can be used to test the quality of soil and to assess the ecology of heavy metal-contaminated and agrochemical-contaminated soils [45,46]. Yan et al. concluded that exogenous bacteria can improve soil enzyme activities in contaminated soils [47]. It has been shown that atrazine has a negative effect on soil enzymes urease, cellulase, catalase, and sucrase, and inoculation with strain ZF-1 alleviated this inhibition [24]. In addition, soil enzymes are widely present in soil and have certain roles, such as urease is involved in the conversion of organic nitrogen; cellulase improves soil fertility and promotes plant growth; and sucrase is closely related to the content of nitrogen, phosphorus, and organic carbon in soil and is a major source of nutrients for both plants and microorganisms [43,48]. The present study showed that strain NM-1 alleviated the inhibition of soil enzymes by prometryn and acetochlor, and the increase in soil enzyme activities indicated that the soil ecosystem was improved, which played a role in repairing the damaged soil environment.

### 3.3. Effect of Strain NM-1 on Soil Bacterial Community Composition

**Figure 4:**
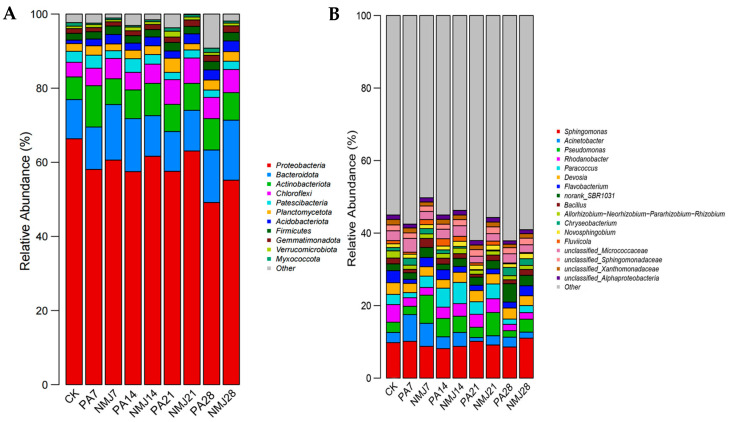
Relative abundance of soil bacterial community at phylum level (**A**) and genus level (**B**).

Figure 4 demonstrates the relative abundance of soil bacteria in different treatment groups at the gate level and genus level. At the gate level, as shown in Figure 4A, the abundance of *Myxococcota* and *Proteobacteria* decreased after the application of prometryn and acetochlor. In contrast, the abundance of Actinobacteriota and Acidobacteriota increased significantly. Wang et al. also suggested that the abundance of Actinobacteriota increased significantly after the addition of acetochlor [49]. This increase can be attributed to the fact that Actinobacteriota have the ability to degrade amide herbicides [42,50]. The addition of strain NM-1 resulted in a significant increase in the abundance of *Proteobacteria*, *Acidobacteriota*, *Gemmatimonadota*, and *Chloroflexi* and a significant decrease in the abundance of *Verrucomicrobiota* and *Planctomycetota*. It has been shown that *Gemmatimonadota* can grow in contaminated soil and has also been found to be positively correlated with vegetation recovery [51]. Therefore, strain NM-1 was able to influence the relative abundance of certain beneficial bacteria in the soil to a certain extent, leading to a more stable and beneficial microbial community in the soil. At the later stage of incubation, the abundance of each phylum stabilized, with *Proteobacteria* (49.16–63.06%), *Bacteroidota* (10.73–16.20%), *Actinobacteriota* (7.26–8.47%), *Chloroflexi* (5.72–6.84%), *Gemmatimonadota* (4.15–5.54%), and *Patescibacteria* (1.96–2.23%) dominated.

As shown in Figure 4B, at the genus level, the abundance of bacteria changed considerably during the incubation process, with a significant increase in the abundance of Sphingomonas, *Bacillus*, *Flavobacterium*, and *Paracoccus* and a significant decrease in the abundance of norank_SBR1031 and Chryseobacterium in the treatment group NMJ. Sphingomonas can grow and improve plant growth under stress conditions such as drought, salinity, and heavy metals in agricultural soils [52]. Inoculation with NM-1 resulted in changes in soil microbial abundance, with an increase in the abundance of beneficial bacteria, which could be stabilized, indicating that NM-1 could restore and maintain soil microbial diversity. The abundance of inoculated *Bacillus* also changed significantly in each group: *Bacillus* accounted for 1.6% of the whole bacterial microbial community in the CK group, and after incubation for a period of time, it accounted for 2.5% in NMJ7, and there was 0.94% in PA7, which indicated that the relative abundance of *Bacillus* was increased due to the incorporation of prometryn and acetochlor providing nutrients for the growth of NM-1. *Bacillus* is an important agrobacterial strain that can adapt to harsh environments and can degrade a wide range of pesticides and other pollutants [36,53,54]. Studies have shown that the addition of the degrading bacteria Chenggangzhangella methanolivorans CHL1 and *Arthrobacter* sp. ART1 to chlorimuron-ethyl and atrazine-contaminated soils significantly alleviated the effects of chlorimuron-ethyl and atrazine on soil microbial biomass, diversity, and community structure [55]. Kong et al. found that exogenous and indigenous bacteria can produce synergistic effects and influence the composition of microbial communities [56]. Therefore, it can be shown that bacterium NM-1 can affect the structure of the soil microbial community and work together with the native microbial community in the remediation of polluted soil.

### 3.4. Effects of Strain NM-1 on Physiological and Ecological Indexes of Soybean Seedlings

**Figure 5:**
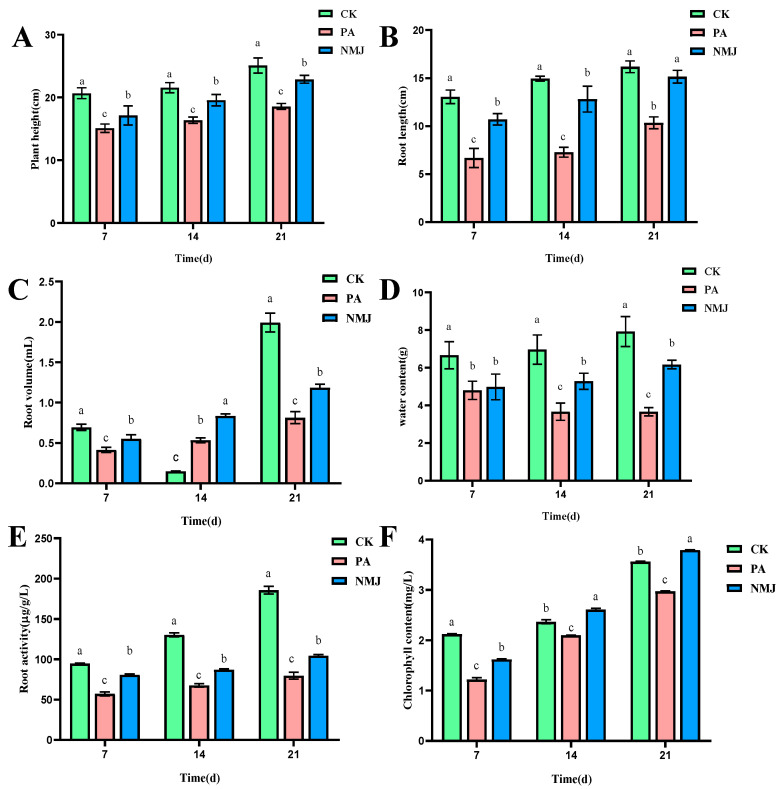
Effects of strain NM-1 on plant height (**A**), root length (**B**), root volume (**C**), water content (**D**), root activity (**E**), and chlorophyll content (**F**) of soybean plants during soil remediation (*p* < 0.05). The error bars represent the standard deviation (SD). Lowercase letters (e.g., a, b, c) above the bars indicate significant differences among different treatment groups at the same time point, as analyzed by one-way analysis of variance (ANOVA). Values marked with different lowercase letters are significantly different (p < 0.05), while those with the same lowercase letter show no significant difference.

In the pot simulation experiment, the effects of different treatments (CK: control group, NM: treatment group applying only prometryn and acetochlor, and NMJ: treatment group applying prometryn and acetochlor and inoculated with strain NM-1) on the growth of soybeans at different times are shown in Figure 5, and the plant height, root length, root volume, water content, chlorophyll content, and root activity of PA in the treatment group were lower than those of the CK group. Some studies have shown that soybeans and corn are seriously damaged in the growth process when the concentration of acetochlor reaches a certain concentration, resulting in dwarfing of seedlings, shorter root lengths, lower fresh weights and reduced chlorophyll content, and even death of seedlings [34,57]. And the growth of *Phaseolus vulgaris* L. seedlings would be inhibited, and the chlorophyll content of seedlings of *Phaeodactylum tricornutum* would be reduced when prometryn reached higher concentrations [5]. However, after the application of strain NM-1, the physiological indexes of the stressed soybean were gradually recovering, and the plant height, root length, root volume, water content, chlorophyll content, and root activity of treatment group NMJ were higher than those of treatment group PA. It is noteworthy that at 21 d, the concentration of chlorophyll in the treatment group NMJ was higher than that in the CK group, and chlorophyll was related to the photosynthesis of plants, and the presence of strain NM-1 promoted the soybean body’s accumulation of organic matter in the soybean [58]. Li et al. also pointed out that inoculation of soil with exogenous strain ACD-9 could promote plant growth and reduce the toxicity of acetochlor to maize plants [59]. Therefore, plant height, root length, root volume, water content, chlorophyll concentration, and root activity of soybean were suppressed after being stressed by prometryn and acetochlor, and inoculation with strain NM-1 alleviated the stress of prometryn and acetochlor on soybean.

### 3.5. Correlation Analysis Between Strain NM-1, Soil Enzymes, Prometryn, Acetochlor, and Bacterial Communities

**Figure 6:**
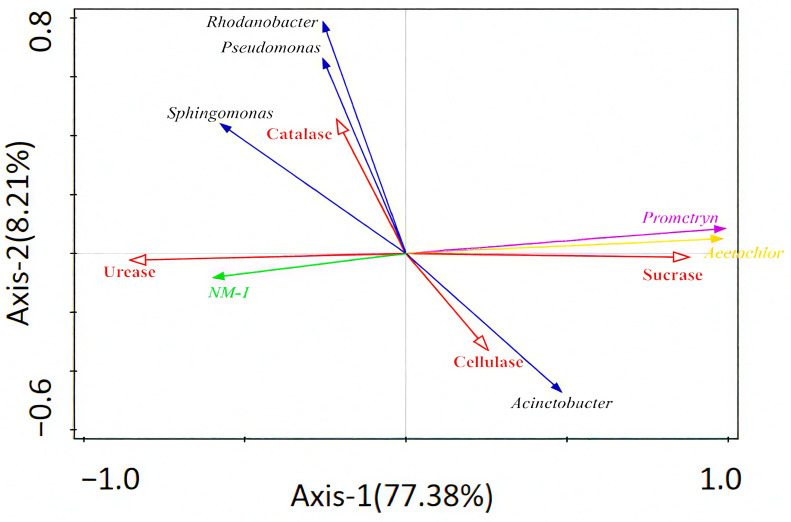
Redundant analysis of relationships between soil enzymes, bacteria, strain NM-1, prometryn, and acetochlor (*p* < 0.05).

Redundancy analysis was used to explore the relationship between strain NM-1 (characterized by its degradation efficiency of prometryn and acetochlor, and its relative abundance in soil), soil enzyme activity, prometryn, acetochlor residues, and bacterial genera (dominant species). Figure 6 shows that Axis 1 and Axis 2 explained 77.38% and 8.21% of the variation, respectively, and accounted for 85.59% of the total variation. Strain NM-1 was positively correlated with *Sphingomonas*, *Rhodanobacter*, and *Pseudomonas* and negatively correlated with *Acinetobacter*, and strain NM-1 was negatively correlated with prometryn and acetochlor residues, positively correlated with catalase and urease, and negatively correlated with cellulase and sucrase. Prometryn and acetochlor residues were positively correlated with cellulase and sucrase, and negatively correlated with the other two enzymes; catalase and urease were negatively correlated with *Acinetobacter* and positively correlated with the other three genera; and the correlation between cellulase and sucrase and the dominant genera of the soil was opposite to that of these two enzymes. Soil microorganisms and soil enzymes interact with each other and work together to maintain the stability of soil microbiota. However, the utility of assessing contaminated soils with several soil enzymes is limited, and some researchers use redundancy analysis (RDA) or Pearson correlation analysis, among other methods, to assess the relationship between soil enzyme activities and contaminated soil environments [60].

### 3.6. Microbe–Enzyme–Plant Correlation Analysis

**Figure 7:**
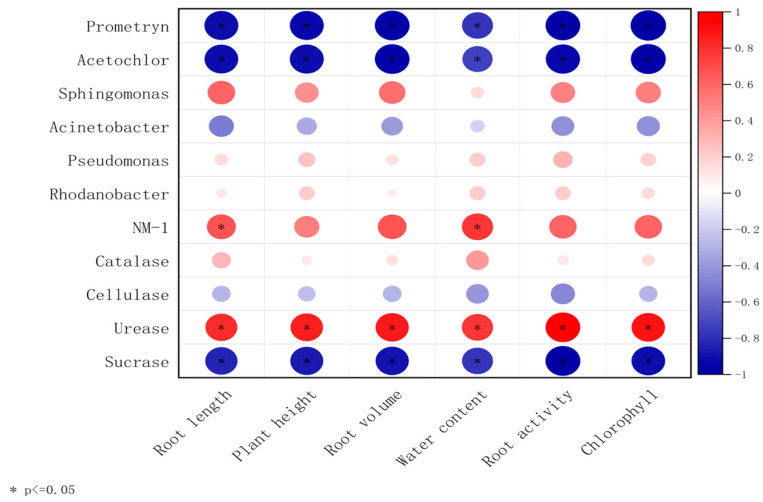
A double-matrix correlation analysis of the relationship between soil enzymes, soil microorganisms, prometryn and acetochlor residues, and soybean plants (*p* < 0.05) (*X*-axis: plant physiological indicators; *Y*-axis: soil dominant bacteria and soil enzymes. Red color represents positive correlation and blue color represents negative correlation.).

The correlation analysis between microbe–enzyme–soybean physiological indicators is shown in Figure 7. Prometryn, acetochlor, sucrase, cellulase, and *Acinetobacter* were negatively correlated with soybean plant height, root length, dry weight, fresh weight, root activity, and chlorophyll content; however, strain NM-1 (characterized by its degradation efficiency of prometryn and acetochlor, and its relative abundance in soil microbial communities); the dominant genera of soil, *Sphingomonas*, *Rhodanobacter*, *Pseudomonas* (characterized by their relative abundance in soil microbial communities, and functional associations with pesticide degradation, soil enzyme activity, and plant growth promotion); and catalase and urease showed the opposite correlations and were positively correlated with soybean plant height, root length, dry weight, fresh weight, root activity, and chlorophyll content. The inoculation of strain NM-1 synergized with microorganisms in the soil and various enzymes to affect the growth of soybean and reduce the damage caused by prometryn and acetochlor stress on soybean.

## 4. Conclusions

To summarize, in this study, *Bacillus* sp NM-1 was used to remove 81.54% of prometryn and 89.47% of acetochlor (100 mg·kg^−1^) from the soil within 15 d, showing good biodegradation characteristics. A comprehensive analysis of the physiological indicators of soybean seedlings, changes in soil enzyme activity, and soil microbial community results showed that strain NM-1 alleviated the stress of prometryn and acetochlor on soil enzymes, and affected the structure and abundance of soil microbial community to a certain extent, and there was a certain correlation between degrading bacterium NM-1, prometryn and acetochlor residues, soil enzyme activity, and soil bacterial community, which influenced each other, resulting in interdependence. Strain NM-1 could prevent damage to soybean seedlings under the stress of prometryn and acetochlor.

## Figures and Tables

**Table 1:** Physical and chemical properties of prometryn and acetochlor.

Properties	Corresponding Characteristics of Prometryn	Corresponding Characteristics of Acetochlor
molecular formula	C_10_H_19_N_5_S	C_14_H_20_ClNO_2_
molecular weight	241.36	269.77
flash point	2 °C	110 °C
boiling point	309.64 °C	134 °C
melting point	118–120 °C	<0 °C
Density	1.157 g/cm^3^	1.1 g/cm^3^
solubility	48.27 mg/L (20 °C)	223 mg/L (25 °C)
Oral LD50 of rats	3150–3750 mg/kg	2148 mg/kg
LD50 of rabbits	>10,200 mg/kg	794 mg/kg

**Table 2:** Biochemical reagents.

Biochemical Reagent	Company	City	Country
2 × Easy Taq Master Mix	Nanjing Novalza Biotechnology Co., Ltd.	Nanjing	China
ClonExpress II One-Step Cloning Kit	Nanjing Novalza Biotechnology Co., Ltd.	Nanjing	China
DL5000 DNA Marker	Nanjing Novalza Biotechnology Co., Ltd.	Nanjing	China
FlyCut^®^ Hind III	NEB (Beijing) Co., Ltd.	Beijing	China
FlyCut^®^ BamH I	NEB (Beijing) Co., Ltd.	Beijing	China
FlyCut^®^ Not I	NEB (Beijing) Co., Ltd.	Beijing	China
Physiological indicator test tube	Huan Kai Technology Company	Guangzhou	China
Soil Enzyme Kit	Huan Kai Technology Company	Guangzhou	China

**Table 3:** Experimental instruments and equipment.

Instrument Name	Manufacturer	City	Country
Electronic balance	Electronic balance Pu Chun Measurement Instrument Co., Ltd.	Shanghai	China
Autoclave	TOMY, Japan	Tokyo	Japan
Constant temperature shaking incubator	Zhicheng Analytical Instrument Manufacturing Co., Ltd.	Shanghai	China
UV-Vis spectrophotometer	Hitachi, Ltd.	Tokyo	Japan
Ultra-micro spectrophotometer	Implen	Munich	Germany
Low-temperature high-speed desktop centrifuge	Beckman Coulter, Inc.	Brea, CA	USA
Vortex mixer	QIAGEN	Hilden	Germany
Bacterial constant temperature incubator	Yiheng Scientific Instruments Co., Ltd.	Shanghai	China
Nucleic acid electrophoresis apparatus	Liuyi Instrument Factory	Beijing	China
PCR machine	Bio-Rad Laboratories, Inc.	Hercules, CA	USA
UV clean bench	Sujing Group Antai Company	Shanghai	China
Rotary evaporator	Shanghai Qiuzuo Scientific Instrument Co., Ltd.	Shanghai	China
pH meter	Bante Instruments Co., Ltd.	Shanghai	China
High-performance liquid chromatography (HPLC)	Agilent Technologies, Inc.	Santa Clara, CA	USA
Automatic snowflake ice machine	Xueke Electric Appliance Co., Ltd.	Ningbo	China
Ultrasonic cell disruptor	Xinzhi Biotechnology Co., Ltd.	Ningbo	China
Digital display constant temperature water bath	Shuoguang Electronic Technology Co., Ltd.	Tianjin	China
Artificial climate chamber	Yiheng Scientific Instruments Co., Ltd.	Shanghai	China

## Data Availability

The original contributions presented in the study are included in the article. Further inquiries can be directed to the corresponding authors.
